# COVID-19 Infection Among Women in Iran Exposed vs Unexposed to Children Who Received Attenuated Poliovirus Used in Oral Polio Vaccine

**DOI:** 10.1001/jamanetworkopen.2021.35044

**Published:** 2021-11-24

**Authors:** Farrokh Habibzadeh, Mohammad M. Sajadi, Konstantin Chumakov, Mahboobeh Yadollahie, Shyamasundaran Kottilil, Ashraf Simi, Kristen Stafford, Saeid Saeidimehr, Mohammad Rafiei, Robert C. Gallo

**Affiliations:** 1 Global Virus Network, Middle East Region, Shiraz, Iran; 2 R&D Headquarters, Petroleum Industry Health Organization, Shiraz, Iran; 3 Institute of Human Virology, University of Maryland School of Medicine, Baltimore; 4 Global Virus Network, Baltimore, Maryland; 5 Office of Vaccines Research and Review, Food and Drug Administration, Global Virus Network Center of Excellence, Silver Spring, Maryland; 6 Petroleum Industry Health Organization, Ahwaz, Iran; 7 Petroleum Industry Health Organization Headquarters, Tehran, Iran

## Abstract

**Importance:**

Live attenuated vaccines may provide short-term protection against infectious diseases through stimulation of the innate immune system.

**Objective:**

To evaluate whether passive exposure to live attenuated poliovirus is associated with diminished symptomatic infection with SARS-CoV-2.

**Design, Setting, and Participants:**

In a longitudinal cohort study involving 87 923 people conducted between March 20 and December 20, 2020, the incidence of COVID-19 was compared between 2 groups of aged-matched women with and without exposure to live attenuated poliovirus in the oral polio vaccine (OPV). Participants were people receiving health care services from the Petroleum Industry Health Organization and residing in 2 cities in Iran (ie, Ahwaz and Shiraz). Participants were women aged 18 to 48 years whose children were aged 18 months or younger and a group of age-matched women from the same residence who had had no potential exposure to OPV.

**Exposures:**

Indirect exposure to live attenuated poliovirus in OPV.

**Main Outcomes and Measures:**

Symptomatic COVID-19, diagnosed by reverse transcription–polymerase chain reaction.

**Results:**

After applying the inclusion and exclusion criteria, 419 mothers (mean [SD] age, 35.5 [4.9] years) indirectly exposed to the OPV and 3771 age-matched women (mean [SD] age, 35.7 [5.3] years) who had no exposure to OPV were available for analysis. COVID-19 was diagnosed in 1319 of the 87 923 individuals in the study population (151 per 10 000 population) during the study period. None of the mothers whose children received OPV developed COVID-19 after a median follow-up of 141 days (IQR, 92-188 days; range, 1-270 days); 28 women (0.74%; 95% CI, 0.47%-1.02%) in the unexposed group were diagnosed with COVID-19 during the 9 months of the study. Point-by-point comparison of the survival curves of the exposed and unexposed groups found that indirect exposure to OPV was significantly associated with decreased COVID-19 acquisition; probability of remaining without infection was 1.000 (95% CI, 1.000-1.000) in the exposed group vs 0.993 (95% CI, 0.990-0.995) in the unexposed group after 9 months (*P* < .001).

**Conclusions and Relevance:**

In this cohort study, indirect exposure to live attenuated poliovirus was associated with decreased symptomatic infection with COVID-19. Further study of the potential protective effect of OPV should be conducted, especially in nations where OPV is already in use for polio prevention and specific COVID-19 vaccines are delayed, less affordable, or fail to meet demand.

## Key Points

**Question:**

Is indirect exposure to live attenuated poliovirus in the oral polio vaccine (OPV) associated with diminished symptomatic infection with SARS-CoV-2?

**Findings:**

In this cohort study of 4190 women in Iran, none of those indirectly exposed to OPV developed COVID-19 during the 9 months of the study, while 0.74% of age-matched women who had no exposure to OPV did develop COVID-19.

**Meaning:**

These findings suggest that indirect exposure to live attenuated poliovirus may be associated with decreased symptomatic COVID-19 infection for at least 6 months.

## Teaser

This cohort study evaluates whether passive exposure to live attenuated poliovirus is associated with diminished symptomatic infection with SARS-CoV-2.

## Introduction

Coronavirus disease 2019 (COVID-19) has affected more than 228 million people worldwide; the death toll exceeded 4.6 million on September 19, 2021.^1^ Currently only a handful of specific vaccines with various degrees of effectiveness have received authorization and are used for immunization in several countries. However, limitations exist in the production and worldwide distribution of the specific vaccines, especially in resource-limited settings. Even for those vaccines with acceptable efficacies, additional studies are needed to assess their long-term effectiveness and safety profile.^2,3,4,5^ An additional concern is the emergence of new antigenic variants of SARS-CoV-2 that may be less sensitive to antibodies induced by the vaccines that were made using the original strain of the virus.^6,7^

A recent study revealed the role of the innate immune system in combating SARS-CoV-2.^8^ It has been suggested that live attenuated vaccines (LAVs) could not only result in long-term specific immunity against their target disease, but also temporarily provide protection against many unrelated infectious diseases through stimulation of the innate immune system.^9,10^ Several controlled trials conducted in 1960s and 1970s on more than 60 000 people revealed that administration of oral polio vaccine (OPV) resulted in an almost 4-fold decrease in the morbidity and mortality associated with influenza with no clinically important attributable adverse effects.^11,12^ Other LAVs, ie, measles and Bacillus Calmette-Guérin vaccines, were also found to produce nonspecific immunity against unrelated diseases.^13,14^ OPV has the advantage of being orally available and having multiple available strains for repeated exposures.

Implementing a large-scale trial to test whether direct exposure to OPV can prevent COVID-19 is not easy, in part because OPV supply is primarily used for the high-priority World Health Organization global polio eradication initiative. However, it is possible to indirectly assess the possibility that OPV has a preventive effect on COVID-19 in countries where the vaccine is still in use.

One of the well-known attributes of OPV is that vaccine recipients shed live virus in their stool, exposing their caregivers and other close household contacts to live attenuated poliovirus.^15^ Theoretically, those with higher chance of fecal exposure (such as mothers of young infants, particularly in countries like Iran, where mothers are almost exclusively taking care of the child) also have higher exposure to live attenuated poliovirus. OPV is a part of the national vaccination program in many countries, including Iran, allowing us to test this hypothesis. According to the national vaccination program in Iran, OPV is given at birth, and at ages 2, 4, 6, and 18 months; a booster dose is given at the age of 6 years.^16^ We conducted this study to test whether mothers with potential indirect exposure to vaccine poliovirus were less likely to develop symptomatic COVID-19 than women without such exposure.

## Methods

### Ethics

The study was conducted in accordance with the Declaration of Helsinki.^17^ The study protocol was approved by the Petroleum Industry Health Organization (PIHO) R&D institutional review board. Data records were coded so that the participants could not be identified by the researchers. All the study participants granted informed consent to use their anonymized data pooled in PIHO databases for this study. This report follows the Strengthening the Reporting of Observational Studies in Epidemiology (STROBE) reporting guideline.

### Setting

This study was conducted between March 20 and December 20, 2020, in the PIHO in Iran, an independent medical center providing health care services free of charge to current employees of the petroleum industry, retirees, and their family members—more than 600 000 people. All necessary health care services, including vaccinations, doctor visits, laboratory tests, or referral to more advanced centers, are provided by the PIHO centers.

### Study Population

A recent study^18^ showed that the incidence of COVID-19 may vary with latitude, temperature, and humidity. We therefore studied all people receiving health care services from PIHO residing in 2 cities in Iran: Ahwaz (65 789 individuals receiving care from the PIHO), with a warm and humid climate, and Shiraz (22 134 individuals receiving care from the PIHO), with a temperate climate.

### Inclusion and Exclusion Criteria

**Figure 1.**
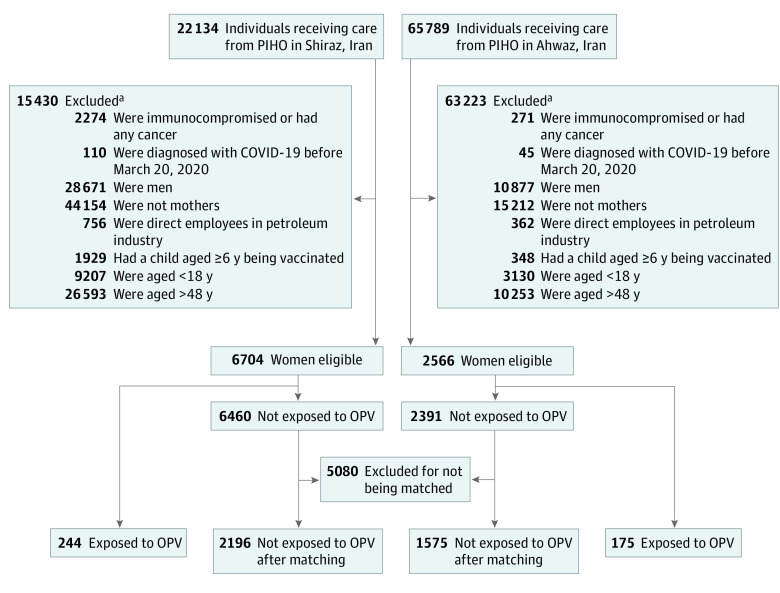
Study Flowchart OPV indicates oral polio vaccine; PIHO, Petroleum Industry Health Organization. ^a^Participants could be excluded for multiple reasons.

All known cases of COVID-19 diagnosed before March 20, 2020, were excluded from the study. In Iran, mothers are the main caregivers of newborn babies (including diaper change) and may have the highest indirect exposure to OPV. Therefore, we excluded other household members who might have variable or limited exposure to attenuated poliovirus shed by a vaccinated child. As the level of maternal exposure to the virus could also vary and depend on the age of the child, we included only those with the highest risk of exposure, ie, mothers aged 18 to 48 years with children aged 18 months or younger; 18 months is the oldest age when a child receives OPV before the booster dose at the age of 6 years, according to the national vaccination protocol in Iran.^16^ Mothers of children aged 6 years were not included in the study because their children no longer needed diaper changes, and the indirect OPV exposure of such mothers was likely limited or questionable. Being employed by the petroleum industry entitles one to use the free health care services. However, by law, children are covered by their fathers’ health insurance (not their mothers’). Women employed by petroleum industry did not necessarily bring their children for vaccination to PIHO centers. Use of the husband’s insurance is legally required, and thus, these children are taken to the health care centers covered by their fathers’ insurance system and not captured in our database. These women were thus excluded from the study. If a member of a family had any type of cancer within 5 years or was immunocompromised (eg, a transplant recipient), the family was also excluded from the study. The children of these families routinely receive inactivated polio vaccine instead of OPV to abolish the risk of infection in immunocompromised person exposed to the live poliovirus in OPV (Figure 1).

### Data Collection

Children aged 18 months and younger who received bivalent OPV (strains 1 and 3) during the study period were identified. The data were retrieved from the PIHO database (updated daily) lists of newborns and children who received OPV during the study period. The vaccines were manufactured by Razi Vaccine and Serum Research Institute. For those who received the vaccine more than once during the study period, the first date was considered for the analysis. Sex, age, residence, number and mean age of the household members, and number of children were used for matching the 2 groups. All variables, as well as the education level of the patients and their spouses, were retrieved from the PIHO databases.

### Exposure and Time of Exposure

All included mothers had no history of clinical COVID-19. In this article, the term *exposure* refers to the participant’s indirect exposure to the live attenuated poliovirus in the OPV. The exposed group included mothers aged 18 to 48 years whose children aged 18 months or younger received at least 1 dose of OPV during the study period. The unexposed group consisted of women who were not exposed to OPV or whose exposure to OPV came after COVID-19 diagnosis. The unexposed group were matched with the exposed group by age, city of residence, number and mean age of the household members, and number of children. In the survival analysis, the time of vaccination was considered the time of the start of follow-up for the exposed group. The unexposed group did not have exposure to the attenuated poliovirus; the time of start of their follow-up was set to the first day of the study.

### Event and Time-to-Event

PIHO has a national surveillance system for the diagnosis and follow-up of patients with COVID-19. All people receiving health care services from PIHO have been invited to complete an electronic form consisting of questions about signs and symptoms for COVID-19. The completed form was then submitted electronically to a center where PIHO staff (usually a nurse) decided whether the person should be further evaluated for COVID-19. If a person was suspected of having COVID-19, he or she was asked to attend a PIHO center to perform chest computed tomography and reverse transcription–polymerase chain reaction (RT-PCR) testing for SARS-CoV-2, performed according to the previously described method.^19^ If they were not referred for further evaluation, the nurse would follow up with them daily for 14 to 21 consecutive days.

In our study, only those with a positive RT-PCR were considered to have COVID-19. We considered occurrence of COVID-19 the event of interest. The time from exposure (beginning of the study for unexposed group and time of OPV vaccination for the exposed group) to the diagnosis of COVID-19 (for those with a positive RT-PCR test) or the end of the study (technically, right-censored) was considered time-to-event.

### Matching

All participants were women. For each mother in the OPV-exposed group, 9 women in the unexposed group were matched for age, residence, number and the mean age of the household members, and number of children. Each woman in the unexposed group was matched only once.

### Statistical Analysis

R software version 4.0.4 (R Project for Statistical Computing) was used for data analysis. Normal probability plot (using geom_qq and stat_qq of the ggplot2 package) was used to determine whether a continuous variable follows normal distribution. Welch *t* test for independent samples (using the t.test function) was used to compare means of 2 normally distributed variables; Wilcoxon rank sum test (using the wilcox.test function) was used to compare not normally distributed data. Matching was done by the matchit function of the MatchIt package.^20^ The nearest method with its default value was used for matching (eAppendix in the Supplement). Kaplan-Meier survival analysis (using Surv and survfit functions of the survival package) was used to study the association of exposure with survival of participants.^21^ Cox regression analysis (using Surv and coxph functions from the same package) was used to determine the association of age, city of residence, number and mean age of household members, and number of children with survival.^22^ The proportional hazards assumption was tested with the cox.zph function (eAppendix in the Supplement). A point-by-point comparison was made at the end of the study between the survival probabilities of the 2 study groups.^23^ *P* < .05 was considered statistically significant, and all tests were 2-tailed. Assuming a baseline event rate of 0.002 events/month in the unexposed group, an exposed-to-unexposed matching ratio of 1:9, an average planned follow-up period of 5 months, a censorship rate of 0.10/month, a 2-sided type I error of less than .05, a study power of greater than 0.90, and a hazard ratio (HR) of less than 0.10, we came to either a minimum sample size of 3086 participants (309 exposed and 2777 unexposed) or occurrence of 22 events during the study.^24^

## Results

A total of 87 923 individuals receiving care at PIHO in Ahwaz and Shiraz were included in the study population. The age distribution of the study population was similar at the 2 study sites (eFigure 1 in the Supplement). The diagnostic protocol (eg, laboratory tests) used for the diagnosis of COVID-19 was uniformly applied to all study participants throughout the PIHO centers. During the 9-month study period, the disease was diagnosed in 1319 individuals (502 [38.1%] women and 817 [61.9%] men), which translates into an incidence rate of 151 per 10 000 population or 17 per 10 000 population per month (12 per 10 000 population for women; 23 per 10 000 for men) per month. The crude incidence rate varied from month to month, with 2 peaks in June to July and October to November (eFigure 2 in the Supplement); incidence was always higher in Ahwaz than Shiraz (median [IQR], 15 [13-24] per 10 000 population in a month vs 10 [4-11] per 10 000 population in a month). None of the study participants had received any specific vaccines against SARS-CoV-2 before or during the entire study period.

**Figure 2.**
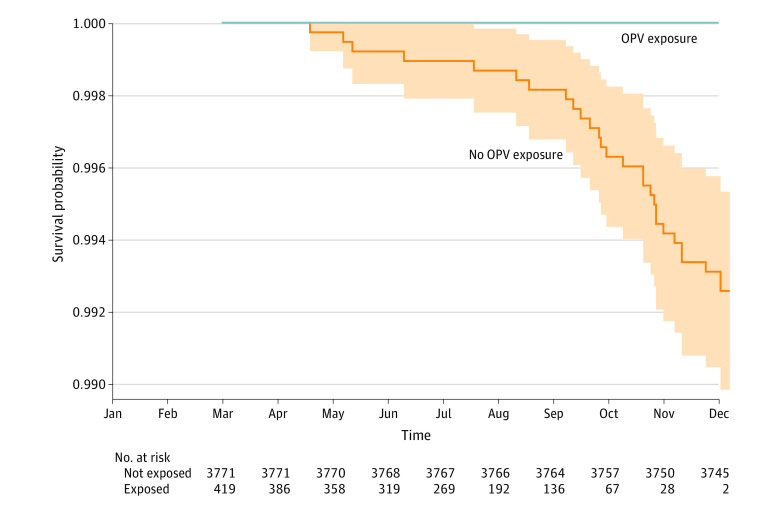
Kaplan-Meier Survival Curves of Mothers With and Without Exposure to Oral Polio Vaccine (OPV) The shaded area represents the 95% CI of the survival in the unexposed group. For the absence of COVID-19 infections in the exposed group, calculation of the interval was not possible for that group. Person-days in the study were 1 016 182 for the unexposed group and 59 068 for the exposed group.

After applying the inclusion and exclusion criteria, 419 women with OPV exposure (244 from Ahwaz and 175 from Shiraz) with a mean (SD) age of 35.5 (4.9) years were left for analysis (Figure 1). These mothers entered the study at various times (when their children received OPV). All were followed up until the end of the study. None developed COVID-19 after a median follow-up of 141 days (IQR, 92-188 days; range, 1-270 days). After matching for age, place of residence, number and the mean age of the household members, and number of children (Table), 3771 women without exposure were left for analysis, of whom 28 (0.74%; 95% CI, 0.47%-1.02%) were diagnosed with COVID-19 during the study period (Figure 2). The minimum time between the last exposure to OPV and the diagnosis of the disease in the 28 mothers with COVID-19, based on the last time their youngest child had received OPV, was 260 days.

**Table.** Measures of Central Tendency and Dispersion of Confounding Variables in the Groups Exposed and Unexposed to the Oral Polio Vaccine, Stratified by Residence Place

Variable	Exposed (n = 419)		Unexposed (n = 3771)			*P* value^a^
	Ahwaz (n = 244)	Shiraz (n = 175)		Ahwaz (n = 2196)	Shiraz (n = 1575)	
**Participant’s age, y**						
Mean (SD)	36.0 (5.0)	34.8 (4.8)		35.6 (5.6)	36.0 (4.7)	.31
Median (IQR)	36.0 (33.0-39.0)	35.0 (31.0-38.0)		35.0 (32.0-39.0)	36.0 (33.0-39.0)	
Range	24.0-48.0	21.0-46.0		19.0-48.0	21.0-48.0	
**Mean age of household members, y**						
Mean (SD)	27.3 (5.8)	26.8 (6.2)		27.3 (5.7)	28.0 (5.3)	.09
Median (IQR)	26.0 (23.5-30.0)	26.0 (22.1-31.0)		26.5 (23.2-30.7)	27.3 (24.3-31.3)	
Range	16.2-47.5	12.0-46.0		13.3-55.3	13.0-57.2	
**No. of household members**						
Mean (SD)	3.8 (1.4)	3.2 (1.0)		3.6 (1.2)	3.2 (0.9)	.20
Median (IQR)	3.0 (3.0-4.0)	3.0 (3.0-4.0)		3.0 (3.0-4.0)	3.0 (3.0-4.0)	
Range	2.0-11.0	1.0-6.0		1.0-13.0	1.0-9.0	
**No. of children**						
Mean (SD)	1.6 (1.2)	1.2 (0.9)		1.5 (1.1)	1.2 (0.8)	.31
Median (IQR)	1.0 (1.0-2.0)	1.0 (1.0-2.0)		1.0 (1.0-2.0)	1.0 (1.0-2.0)	
Range	0.0-7.0	0.0-4.0		0.0-10.0	0.0-3.0	

^a^ Welch *t* test for independent samples comparing means of the exposed and unexposed groups.

**Figure 3.**
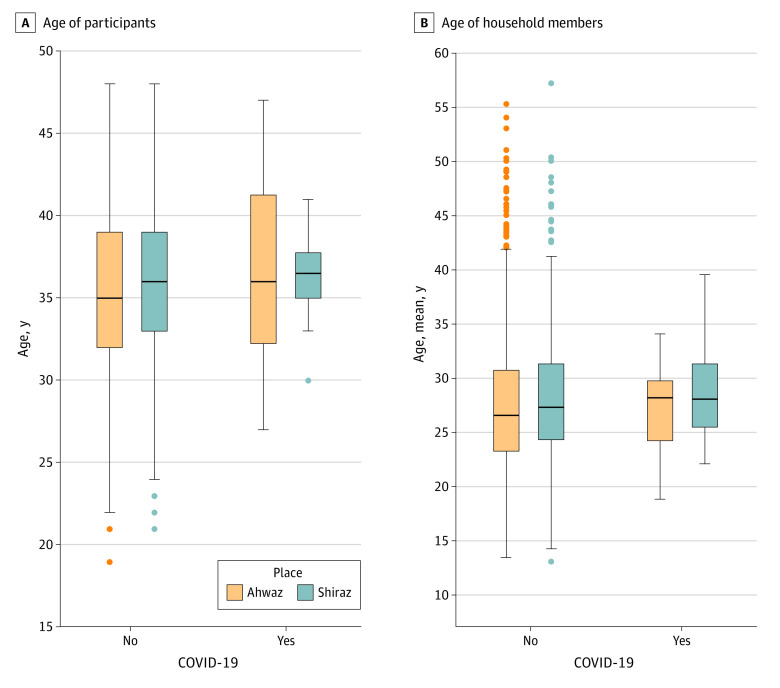
Age of Study Participants and the Mean Age of Household Members, Stratified by the Disease Condition and the Residence Place The horizontal line in the middle of each box indicates the median, the top and bottom borders of the box mark the 75th and 25th percentiles, respectively. The upper whisker indicates the largest data point within 1.5 times the IQR greater than the 75th percentile; the lower whisker indicates the smallest point within 1.5 times the IQR less than the 25th percentile. Points represent outliers.

**Figure 4.**
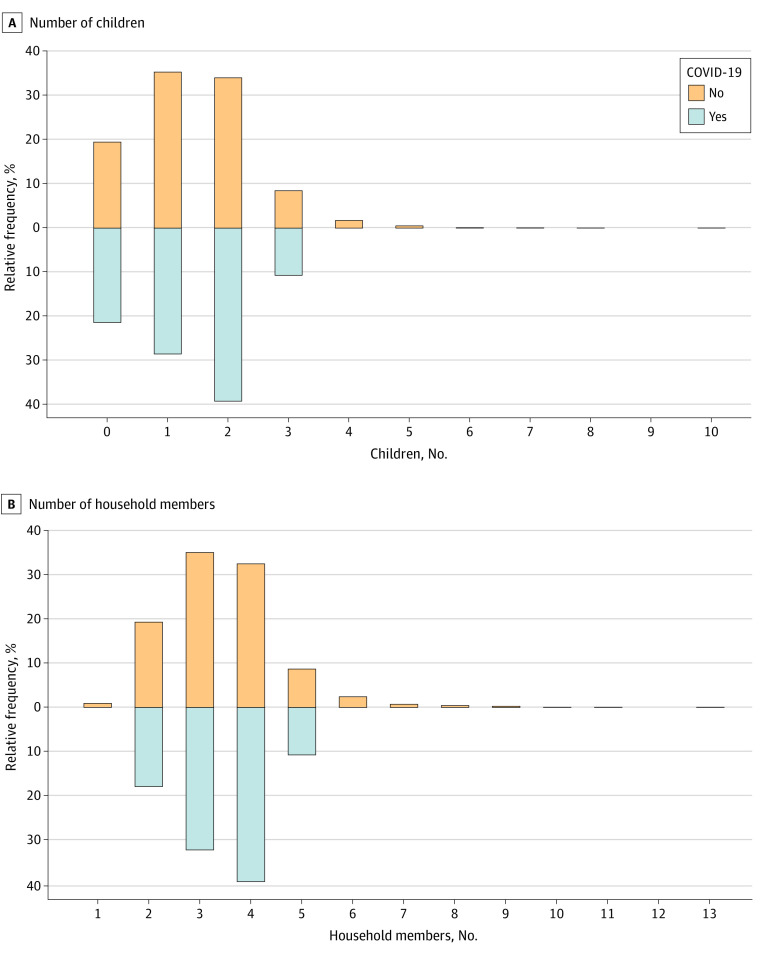
Distribution of the Number of Children and Household Members in the Studied Participants, Stratified by the Disease Status None of the participants with COVID-19 had 4 or more children; 0.08% of those without COVID-19 had 6 children; 0.05%, 7 children; 0.03%, 8 children; 0, 9 children; and 0.03%, 10 children. None of the participants with COVID-19 had 6 or more household members; 0.43% of those without COVID-19 had 8 household members; 0.27%, 9 household members; 0.03%, 10 household members; 0.03%, 11 household members; 0, 12 household members; and 0.03%, 13 household members.

All study participants were vaccinated with OPV during their childhood according to the national vaccination program in Iran.^16^ The distribution of the participants’ age was normal; those of other confounders were not (eFigure 3 in the Supplement). The distribution of confounding variables was not significantly different between women with and without COVID-19 in the unexposed group (Figure 3 and Figure 4). Patients with COVID-19 were educated for a mean (SD) of 14.5 (3.3) years; it was a mean (SD) of 15.5 (2.4) years for their spouses.

The number of children and the number of household members had a high correlation (Spearman ρ, 0.96). Therefore, the number of children was not included in the Cox regression. The proportional hazard assumption was met by all the covariates in the model (age, place of residence, number and the mean age of the household members, and exposure to OPV). The HR derived from the model was not significantly different from 1 for age (HR, 1.03; 95% CI, 0.94-1.13), number of household members (HR, 0.97; 95% CI, 0.63-1.49), mean age of household members (HR, 1.01; 95% CI, 0.93 to 1.09), and residence place (living in Shiraz: HR, 1.36; 95% CI, 0.64 to 2.90); none of them were associated with the survival probability (eAppendix in the Supplement).

Absence of COVID-19 infections in the exposed group made it impossible to accurately calculate the HR for the exposure to OPV; it was estimated at 1 × 10^−7^ (95% CI, 0 to ∞). Point-by-point comparison of the survival probabilities revealed a significant (*P* < .001) difference between the 2 groups after 9 months; with a probability of 1.000 (95% CI, 1.000-1.000) in the exposed group vs 0.993 (95% CI, 0.990-0.995) in the unexposed group (Figure 2).

## Discussion

In this study, none of the mothers whose infants received OPV developed COVID-19. The incidence of COVID-19 was relatively low in early months of the study (eFigure 2 in the Supplement). The Persian new-year festival, which coincided with the beginning of the study, may have been an important factor associated with this relatively low incidence. Last year, most Iranians observed the strict hygienic rules set by the national health authorities for several weeks (particularly in March and April 2020). These were associated with a decrease in the rate of infection. When incidence increased again, the disease affected older individuals more frequently.^25^ Nonetheless, none of our study participants were older than 48 years. That might explain the low incidence of the disease among our participants during the early weeks of the study (Figure 2). However, changes in the rate of infection equally affected both exposed and unexposed groups.

The uniform diagnostic protocol made it possible to believe that the affected people in both study groups could be identified with equal likelihood. Most of the women in the unexposed group developed COVID-19 during October and November (Figure 2), the months with the highest incidence rate of the disease (eFigure 2 in the Supplement).

Several risk factors have been associated with the acquisition of COVID-19. A systematic review^26^ showed that limited access to health care services is associated with higher rates of the infection. PIHO provides free quality health care services with almost the same standards throughout the country.^27^ Therefore, the level of access of study participants was similar in both study groups.

Another factor affecting the infection rate is family income.^25^ Income, however, might be considered a surrogate index for the level of access to health care services. We could not assess the income of the study participants. Nonetheless, considering their ready access to free health care services, income might not be an important risk factor for acquisition of COVID-19 in our study. Furthermore, income may be associated with education.^28^ The women in the unexposed groups with COVID-19 and their spouses had middle to high levels of education, and we assumed these participants had middle to high income.

Other contributing factors for infection rates include high volume of tourism, international trade, urbanization, and high population density.^26^ US sanctions on Iran as well as the pandemic have markedly decreased the international trade and number of tourists. Sanctions have affected parts of the health sector^29^; however, the participants in each study group were equally affected.

The level of urbanization and the population density mostly depend on the city of residence; the mothers in the exposed group were matched with unexposed women in the same city. We did not assess whether the residence neighborhoods of the participants in the studied cities were substantially different. However, we matched 9 women in the unexposed group to each mother in the exposed group according to several confounders; it is unlikely that most of these matched women came from a part of the city with very different incidence rates compared with the mother in the exposed group. Furthermore, the study groups were matched for the mean number of household members and the number of children, both, to some extent, reflecting population density (Figure 4). The study groups were also matched for the participants’ age (Figure 3) and sex, the 2 variables considered important factors for the infection rate.^25^

The last time a patient in our series was exposed to OPV was more than 260 days before their COVID-19 diagnosis, suggesting that exposure to OPV may protect people against SARS-CoV-2 for at least 6 months. We did not assess the previous exposure to OPV among the 3743 women in the unexposed group who had not developed the disease during the study period. Even if they had an exposure to OPV during the few weeks before the study, they would have been categorized in the exposed group. This would increase the sample size of mothers with OPV exposure who did not develop COVID-19, further supporting our findings and our hypothesis that exposure to OPV may be partially protective against COVID-19.

Although the mechanism of nonspecific protective effects of OPV against unrelated infections is unknown, it may be related to the reprogramming of innate immune system (also known as trained immunity).^9,30,31^ LAVs induce trained immunity through complex carefully orchestrated interactions among immunological signals, certain cell metabolites, and epigenetic reprogramming.^31^ Cell metabolism changes to produce enough energy and precursors necessary for modulation of the epigenetic process involved.^32,33,34^ The architecture of the chromatin regions encoding for genes required for host defense is also changed.^35^ These regions are normally highly condensed in unstimulated cells. Primary stimulation of cells with LAVs (eg, OPV) results in the previously mentioned changes. These lead to deposition of chromatin marks, altered status of the DNA methylation, and methylation and acetylation of nuclear histones, with resultant unfolding of chromatin regions that enhances transcription and gene expression of factors involved in antiviral defense. The changes are only partially removed after the primary stimulus is eliminated. Nonetheless, the remaining changes may be enough to result in a strong, rapid immune response of the stimulated cells to challenge secondary stimuli (eg, SARS-CoV-2).^31^

### Limitations

This study has limitations. First, the mothers in the OPV-exposed group, who were caring for an infant or young child, might have spent more time at home and hence had less exposure to SARS-CoV-2. We did not measure the time each study participant spent at home. Some of these mothers might indeed be even at higher levels of exposure. As part of Iranian ceremonies, they may have been visited by numerous friends and relatives who came to meet the newborn.

Additionally, the strict case definition of COVID-19 in our study (considering only those with a positive RT-PCR test as having COVID-19) made our case selection very specific.^36^ We cannot be sure whether any of the exposed women in our study were not infected, nor can we exclude the possibility that we missed patients with asymptomatic infection or a very mild form of the disease because we only tested patients with clinical symptoms.

## Conclusions

This study is an initial observation of potential interaction between OPV and risk of COVID-19. In this cohort study, we found that exposure to OPV was associated with protection against SARS-CoV-2 infection for at least 6 months. If exposure to attenuated poliovirus could prevent SARS-CoV-2 infection, then vaccination with OPV could break the chain of transmission in the community. This would be particularly important for countries that already use OPV for immunization and where coverage for COVID-19 vaccination is still low.

## Notes

Supplement. **eFigure 1.** Age Distribution of the Study Population Stratified by Residence Place **eFigure 2.** Number of New Cases of COVID-19 per 10 000 People Reported During the Study Period, Stratified by Residence Place **eFigure 3.** QQ-Plots of the 4 Studied Continuous Variables **eAppendix. **Supplementary Methods
