# Error in Figure

**DOI:** 10.1001/jamanetworkopen.2021.43970

**Published:** 2021-12-10

In the Original Investigation titled “COVID-19 Infection Risk Among Women in Iran Exposed vs Unexposed to Children Who Received Attenuated Poliovirus Used in Oral Polio Vaccine,”^1^ published November 24, 2021, there was an error in Figure 1. Of the 65 789 individuals who received care in Ahwaz, Iran, 59 085 were excluded, and of the 22 134 individuals who received care in Shiraz, Iran, 19 568 were excluded. This article has been corrected.^1^
