# Supplementary material for: COVID-19 Infection Among Women in Iran Exposed vs Unexposed to Children Who Received Attenuated Poliovirus Used in Oral Polio Vaccine
Source: JAMA Netw Open. 2021 Nov 24;4(11):e2135044. doi: 10.1001/jamanetworkopen.2021.35044 (PMC8613592; doi:10.1001/jamanetworkopen.2021.35044)
Supplement: Supplement. — eFigure 1. Age Distribution of the Study Population Stratified by Residence Place eFigure 2. Number of New Cases of COVID-19 per 10 000 People Reported During the Study Period, Stratified by Residence Place eFigure 3. QQ-Plots of the 4 Studied Continuous Variables eAppendix. Supplementary Methods [file jamanetwopen-e2135044-s001.pdf]

Supplemental Online Content

Habibzadeh F, Sajadi MM, Chumakov K, et al. COVID-19 infection among women in Iran exposed vs unexposed to children who received attenuated poliovirus used in oral polio vaccine. *JAMA Netw Open.* 2021;4(11):e2135044.
doi:10.1001/jamanetworkopen.2021.35044

eFigure 1. Age Distribution of the Study Population Stratified by Residence Place

eFigure 2. Number of New Cases of COVID-19 per 10 000 People Reported During the Study Period, Stratified by Residence Place

eFigure 3. QQ-Plots of the 4 Studied Continuous Variables

eAppendix. Supplementary Methods

This supplemental material has been provided by the authors to give readers additional information about their work.

© 2021 Habibzadeh F et al. *JAMA Network Open.*

eFigure 1. Age Distribution of the Study Population Stratified by Residence Place

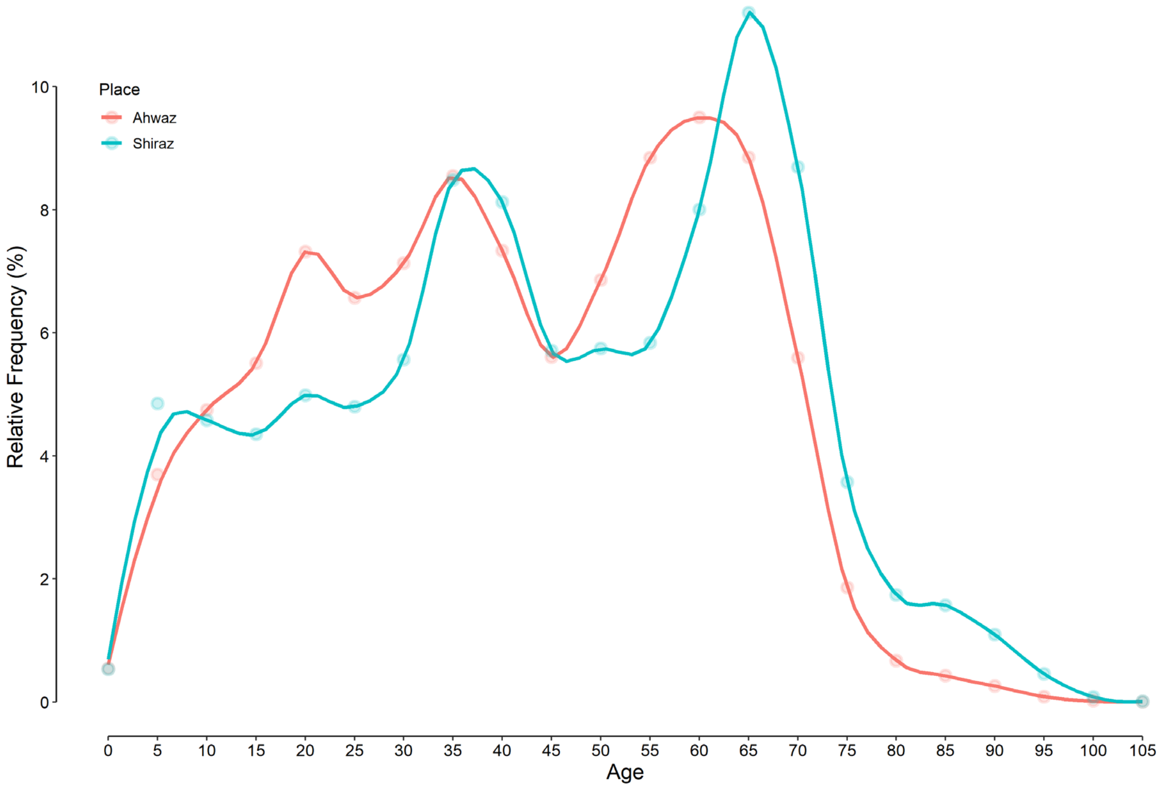

© 2021 Habibzadeh F et al. *JAMA Network Open.*

eFigure 2. Number of New Cases of COVID-19 per 10 000 People Reported During the Study Period, Stratified by Residence Place

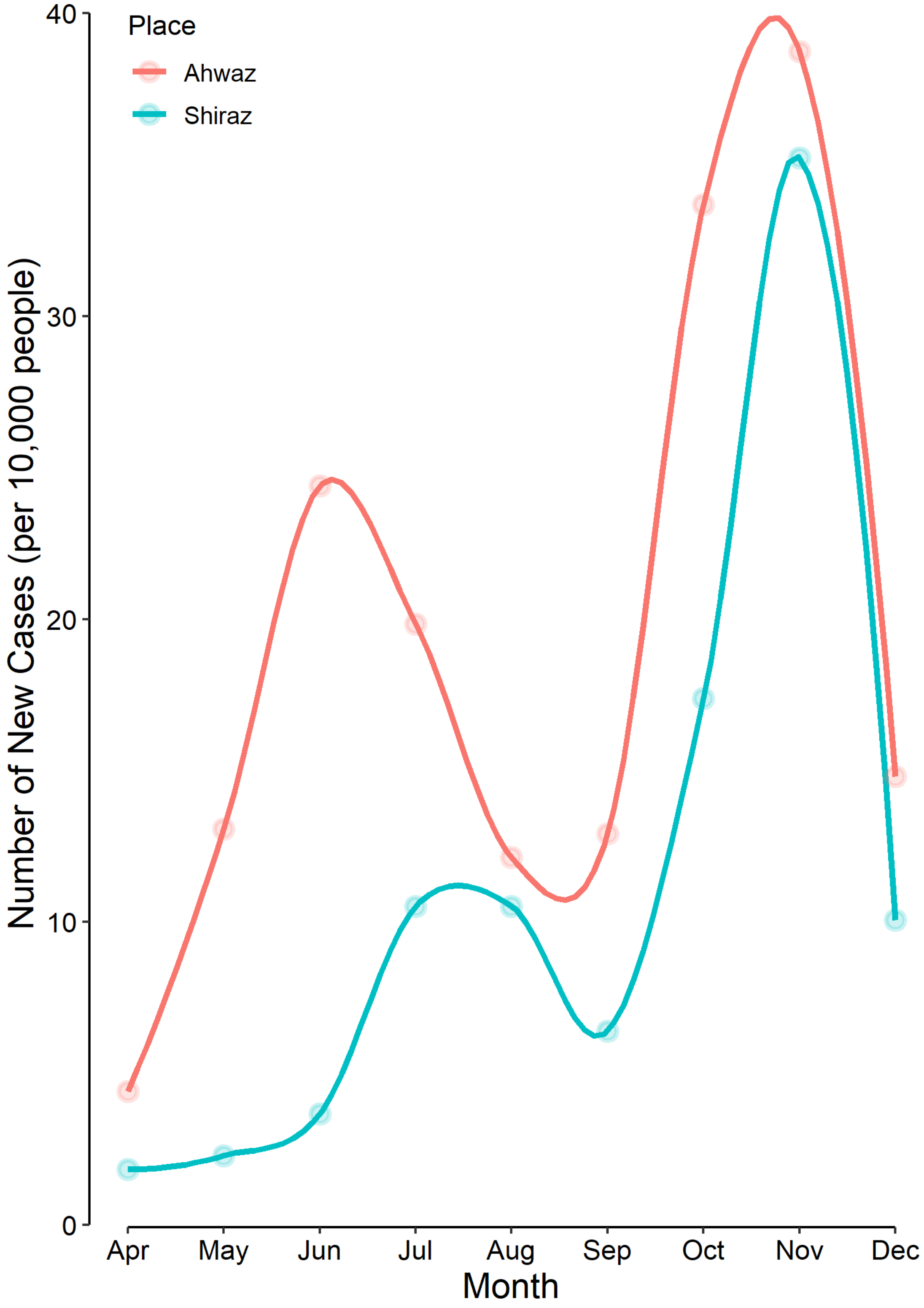

© 2021 Habibzadeh F et al. *JAMA Network Open.*

eFigure 3. QQ-Plots of the 4 Studied Continuous Variables

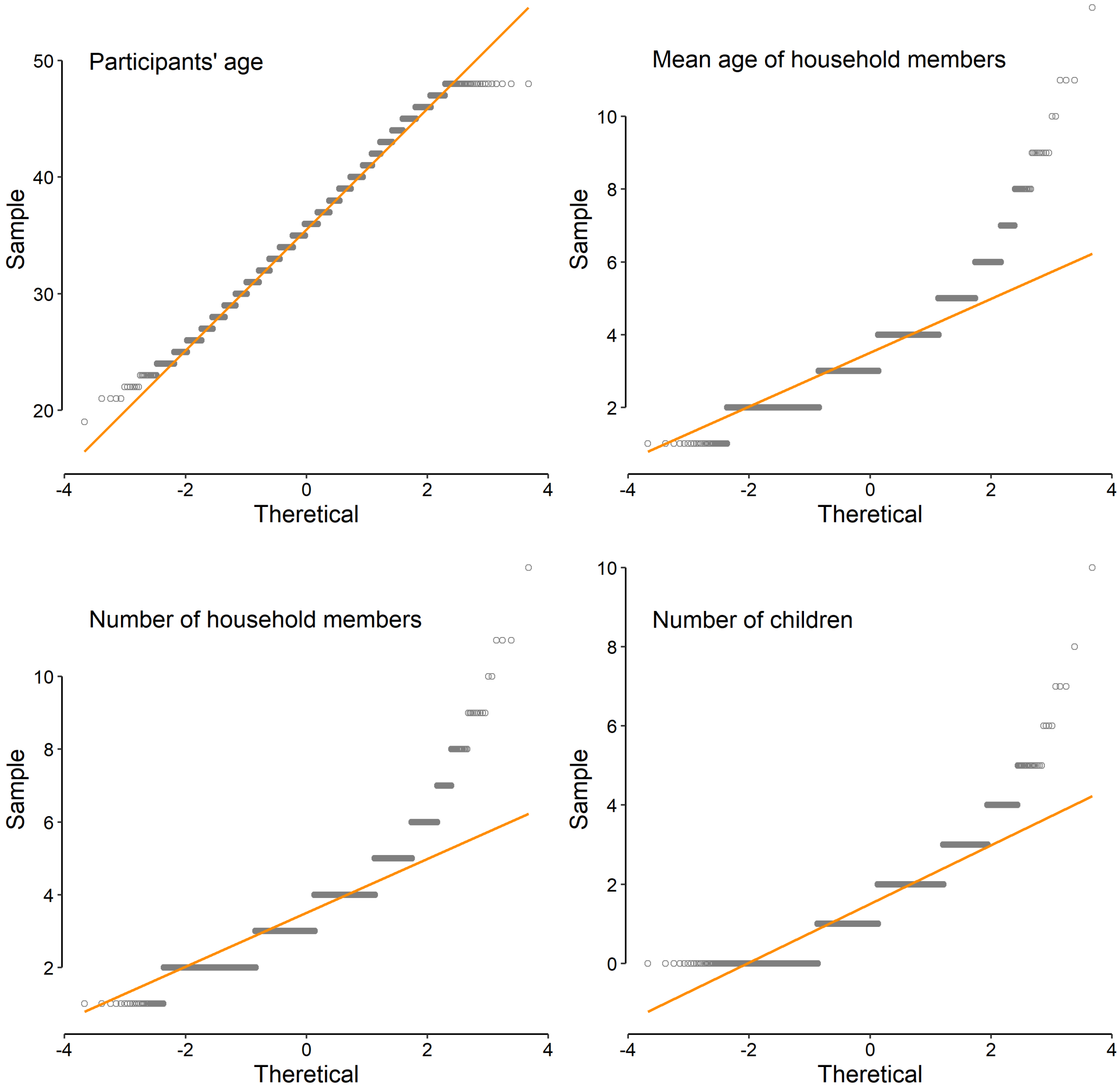

© 2021 Habibzadeh F et al. *JAMA Network Open.*

eAppendix. Supplementary Methods

Data dictionary

Place	Residence city (Ahwaz or Shiraz)	Age	Participants' age (years)
Time	time-to-event (days)	meanAge	Mean age of the household members
COVID	Disease status	n	Number of the household members
Rx	Exposure status, exposed or unexposed	childNum	Number of children

Welch *t* test for independent groups

t.test(dd$Age ~ dd$Rx)   # comparing means of participants' age in exposed and unexposed groups

```
        Welch Two Sample t-test

data:  dd$Age by dd$Rx
t = 1.0275, df = 528.78, p-value = 0.3046
alternative hypothesis: true difference in means is not equal to 0
95 percent confidence interval:
 -0.2401112  0.7667620
sample estimates:
mean in group Not exposed     mean in group Exposed
         35.71917                  35.45585
```

Matching

```
d %>%                                  # d contains the data for all study population
  filter(Place == "Ahwaz") -> d_Ahwaz
d %>%
  filter(Place == "Shiraz") -> d_Shiraz

ratio = 9
matchit(formula = as.logical(Rx) ~ Age + meanAge + childNum +
        n, data = d_Ahwaz, method = "nearest", replace = FALSE, ratio = ratio)
```

```
Summary of Balance for All Data:
         Means Treated Means Control Std. Mean Diff. Var. Ratio eCDF Mean
distance        0.0579        0.0356          0.3812     4.6736    0.1877
Age            35.9549       39.2508         -0.6635     0.6846    0.1156
meanAge        27.2583       31.0691         -0.6572     0.6669    0.1104
childNum        1.6270        1.5268          0.0870     1.2267    0.0165
n               3.7500        3.5551          0.1370     1.5462    0.0209
         eCDF Max
distance   0.3253
Age        0.2754
meanAge    0.3082
childNum   0.0478
n          0.0531


Summary of Balance for Matched Data:
         Means Treated Means Control Std. Mean Diff. Var. Ratio eCDF Mean
distance        0.0579        0.0541          0.0643     3.0685    0.0010
Age            35.9549       35.5364          0.0842     0.7781    0.0204
meanAge        27.2583       27.3047         -0.0080     1.0189    0.0130
childNum        1.6270        1.5168          0.0956     1.0807    0.0113
n               3.7500        3.5774          0.1213     1.3065    0.0145
         eCDF Max Std. Pair Dist.
distance   0.0137          0.0669
Age        0.0578          0.6786
meanAge    0.0606          0.5158
childNum   0.0524          0.8246
n          0.0533          0.7443

Percent Balance Improvement:
         Std. Mean Diff. Var. Ratio eCDF Mean eCDF Max
distance            83.1       27.3      99.4     95.8
Age                 87.3       33.8      82.3     79.0
```

© 2021 Habibzadeh F et al. *JAMA Network Open.*

```
meanAge             98.8       95.4      88.2      80.3
childNum            -9.9       62.0      31.1      -9.6
n                   11.4       38.7      30.5      -0.4

Sample Sizes:
          Control Treated
All          6460     244
Matched      2196     244
Unmatched    4264       0
Discarded       0       0

Percent Balance Improvement:
         Std. Mean Diff. Var. Ratio eCDF Mean eCDF Max
distance            83.1       27.3      99.4      95.8
Age                 87.3       33.8      82.3      79.0
meanAge             98.8       95.4      88.2      80.3
childNum            -9.9       62.0      31.1      -9.6
n                   11.4       38.7      30.5      -0.4

Sample Sizes:
          Control Treated
All          6460     244
Matched      2196     244
Unmatched    4264       0
Discarded       0       0
```

matchit(formula = as.logical(Rx) ~ Age + meanAge + childNum +
n, data = d_Shiraz, method = "nearest", replace = FALSE, ratio = ratio)

```
Summary of Balance for All Data:
         Means Treated Means Control Std. Mean Diff. Var. Ratio eCDF Mean
distance        0.1104        0.0651          0.6788     1.6780    0.2078
Age            34.7600       38.6692         -0.8077     0.7181    0.1396
meanAge        26.8134       30.9354         -0.6606     0.7909    0.1196
childNum        1.2171        1.3739         -0.1657     1.1109    0.0297
n               3.1886        3.4203         -0.2225     0.9820    0.0274
         eCDF Max
distance   0.3457
Age        0.3046
meanAge    0.2708
childNum   0.1374
n          0.1530

Summary of Balance for Matched Data:
         Means Treated Means Control Std. Mean Diff. Var. Ratio eCDF Mean
distance        0.1104        0.0886          0.3263     1.8878    0.0530
Age            34.7600       35.9740         -0.2508     1.0692    0.0434
meanAge        26.8134       28.0075         -0.1914     1.4112    0.0404
childNum        1.2171        1.2362         -0.0201     1.2807    0.0220
n               3.1886        3.2254         -0.0354     1.2384    0.0188
         eCDF Max Std. Pair Dist.
distance   0.1879          0.3296
Age        0.1098          0.7168
meanAge    0.1479          0.7293
childNum   0.0698          0.8979
n          0.0717          0.8924

Percent Balance Improvement:
         Std. Mean Diff. Var. Ratio eCDF Mean eCDF Max
distance            51.9      -22.8      74.5      45.6
Age                 68.9       79.8      68.9      63.9
meanAge             71.0      -46.9      66.2      45.4
childNum            87.8     -135.2      26.1      49.2
n                   84.1    -1075.3      31.6      53.1

Sample Sizes:
          Control Treated
All          2391     175
Matched      1575     175
Unmatched     816       0
Discarded       0       0
```

Kaplan-Meier survival plot (Fig 2)

© 2021 Habibzadeh F et al. *JAMA Network Open.*

```
fit <- survfit(formula = Surv(Time, as.integer(COVID)) ~ Rx, data = dd, type = "kaplan-meier")
# dd containes the matched study participants
```

```
                Rx=Not exposed
 time n.risk n.event survival  std.err lower 95% CI upper 95% CI
   48   3771       1    1.000 0.000265        0.999        1.000
   66   3770       1    0.999 0.000375        0.999        1.000
   71   3769       1    0.999 0.000459        0.998        1.000
   99   3768       1    0.999 0.000530        0.998        1.000
  137   3767       1    0.999 0.000593        0.998        1.000
  160   3766       1    0.998 0.000649        0.997        1.000
  167   3765       1    0.998 0.000701        0.997        1.000
  187   3764       1    0.998 0.000749        0.996        0.999
  191   3763       1    0.998 0.000795        0.996        0.999
  195   3762       1    0.997 0.000837        0.996        0.999
  200   3761       1    0.997 0.000878        0.995        0.999
  205   3760       1    0.997 0.000917        0.995        0.999
  206   3759       1    0.997 0.000954        0.995        0.998
  209   3758       1    0.996 0.000990        0.994        0.998
  218   3757       1    0.996 0.001025        0.994        0.998
  229   3756       2    0.995 0.001091        0.993        0.998
  233   3754       1    0.995 0.001122        0.993        0.997
  235   3753       1    0.995 0.001153        0.993        0.997
  236   3752       2    0.994 0.001212        0.992        0.997
  240   3750       1    0.994 0.001240        0.992        0.997
  246   3749       1    0.994 0.001268        0.991        0.996
  250   3748       2    0.993 0.001322        0.991        0.996
  263   3746       1    0.993 0.001347        0.990        0.996
  271   3745       2    0.993 0.001398        0.990        0.995

                Rx=Exposed
     time n.risk n.event survival std.err lower 95% CI upper 95% CI
```

```
ds <- data.frame(time = fit$time, n.risk = fit$n.risk, n.event = fit$n.event,
                 n.censor = fit$n.censor, surv = fit$surv, upper = fit$upper, lower = fit$lower)

autoplot(fit, censor.shape = '', conf.int.alpha = 0.1) +
  scale_x_continuous(breaks=seq(0, duration, 30), labels = c("Mar", "Apr", "May", "Jun", "Jul",
                                                             "Aug", "Sep", "Oct", "Nov", "Dec")) +
  scale_y_continuous(breaks=seq(0.990, 1.00, 0.002)) +
  theme_classic() +
  labs(x = "Time", y = "Survival Probability") +
  theme(panel.grid.major.y = element_blank(), panel.grid.minor.y = element_blank(),
     panel.grid.major.x = element_blank(), panel.grid.minor.x = element_blank(),
     axis.text = element_text(size = rel(1), color = "black"),
     axis.title = element_text(size = rel(1.4)),
     legend.position = c(0.03, 0.05),
     legend.justification = c("left", "bottom"), aspect.ratio = 5/4,
     legend.text = element_text(size = rel(0.9))) +
  guides(x = "axis_truncated", y = "axis_truncated") +
  guides(color = guide_legend(title = "OPV"), fill = guide_legend(title = "OPV"))
```

Cox regression analysis

```
res.cox <- coxph(Surv(Time, as.integer(COVID)) ~ Rx + Age + Place + n + meanAge, data = dd)
```

```
  n= 4190, number of events= 28

                  coef  exp(coef)   se(coef)      z Pr(>|z|)
RxYes       -1.613e+01  9.920e-08  3.567e+03 -0.005    0.996
Age          2.844e-02  1.029e+00  4.609e-02  0.617    0.537
PlaceShiraz  3.087e-01  1.362e+00  3.865e-01  0.799    0.424
n           -3.363e-02  9.669e-01  2.195e-01 -0.153    0.878
meanAge      5.999e-03  1.006e+00  3.923e-02  0.153    0.878

            exp(coef) exp(-coef) lower .95 upper .95
```

© 2021 Habibzadeh F et al. *JAMA Network Open.*

```
RxYes       9.920e-08  1.008e+07    0.0000       Inf
Age         1.029e+00  9.720e-01    0.9400     1.126
PlaceShiraz 1.362e+00  7.344e-01    0.6384     2.904
n           9.669e-01  1.034e+00    0.6289     1.487
meanAge     1.006e+00  9.940e-01    0.9316     1.086

Concordance= 0.582  (se = 0.05 )
Likelihood ratio test= 2.88  on 5 df,   p=0.7
Wald test            = 1.38  on 5 df,   p=0.9
Score (logrank) test = 2.16  on 5 df,   p=0.8
```

Test of proportional hazards assumption

test.ph <- cox.zph(res.cox, transform="identity")

```
           chisq df    p
Rx      6.73e-08  1 1.00
Age     3.68e-01  1 0.54
Place   2.34e-01  1 0.63
n       2.36e-01  1 0.63
meanAge 1.42e-01  1 0.71
GLOBAL  1.64e+00  5 0.90
```

© 2021 Habibzadeh F et al. *JAMA Network Open.*
